# A signature of 33 immune‐related gene pairs predicts clinical outcome in hepatocellular carcinoma

**DOI:** 10.1002/cam4.2921

**Published:** 2020-02-18

**Authors:** Xiao‐Yan Sun, Shi‐Zhe Yu, Hua‐Peng Zhang, Jie Li, Wen‐Zhi Guo, Shui‐Jun Zhang

**Affiliations:** ^1^ Department of Hepatobiliary and Pancreatic Surgery The First Affiliated Hospital of Zhengzhou University Zhengzhou Henan China; ^2^ Open and Key Laboratory of Hepatobiliary & Pancreatic Surgery and Digestive Organ Transplantation at Henan Universities Zhengzhou Henan China; ^3^ Henan Key Laboratory of Digestive Organ Transplantation Zhengzhou Henan China

**Keywords:** gene pairs, HCC, prognosis, tumor immunology

## Abstract

**Objective:**

Hepatocellular carcinoma (HCC) has become the second most common tumor type that contributes to cancer‐related death worldwide. The study aimed to establish a robust immune‐related gene pair (IRGP) signature for predicting the prognosis of HCC patients.

**Methods:**

Two RNA‐seq datasets (The Cancer Genome Atlas Program and International Cancer Genome Consortium) and one microarray dataset (http://www.ncbi.nlm.nih.gov/geo/query/acc.cgi?acc=GSE14520) were included in this study. We used a series of immune‐related genes from the ImmPort database to construct gene pairs. Lasso penalized Cox proportional hazards regression was employed to develop the best prognostic signature. We assigned patients into two groups with low immune risk and high immune risk. Then, the prognostic ability of the signature was evaluated by a log‐rank test and a Cox proportional hazards regression model.

**Results:**

After 1000 iterations, the 33‐immune gene pair model obtained the highest frequency. As a result, we chose the 33 immune gene pairs to establish the immune‐related prognostic signature. As we expected, the immune‐related signature accurately predicted the prognosis of HCC patients, and high‐risk groups showed poor prognosis in the training datasets and testing datasets as well as in the validation datasets. Furthermore, the immune‐related gene pair (IRGP) signature also showed higher predictive accuracy than three existing prognostic signatures.

**Conclusion:**

Our prognostic signature, which reflects the link between the immune microenvironment and HCC patient outcome, is promising for prognosis prediction in HCC.

## Graphical abstract

We used a series of immune‐related genes to construct an immune‐related gene pair. Then the lasso‐penalized Cox proportional hazards regression was applied to develop the best prognosis signature. Finally, we validated our immune‐related gene pair signature.

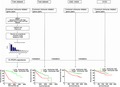

## 1 INTRODUCTION

Hepatocellular carcinoma has been recognized as the fifth most common primary malignant tumor and the second leading cause of cancer‐related deaths globally.^1^ The main risk factor for tumorigenesis is chronic viral hepatitis, alcoholic liver disease, diabetes and nonalcoholic steatohepatitis (NASH).^2^ The outcome of HCC is poor: according to the Surveillance, Epidemiology, and End Results (SEER) database, the 5‐year survival rate of local hepatocellular carcinoma patients is 30.5%, and the rate is less than 5% for patients with distant metastasis.^3^ Although partial hepatectomy and liver transplantation are the main treatment methods for early‐stage patients, few patients are eligible for these treatments, and approximately 70% of patients will relapse within five years after surgery.^4^ Moreover, it is generally observed that HCC is not very sensitive to radiation and chemotherapy. To date, sorafenib and lenvatinib have been approved as targeted therapies for hepatocellular carcinoma by the United States Food and Drug Administration (FDA) to treat unresectable HCC; however, they have limited effectiveness.

It had been shown that several components of the immune system were key factors during tumor development and progression. Recent studies also indicated that dysregulation of the immune system including alteration in the number or function of immune cells, the release of chemokine and cytokine, and expression of inhibitory receptors or their ligands can lead to the progression of hepatocellular carcinoma.^5^, ^6^ Moreover, immune checkpoint inhibitors that specifically target PD1/PD‐L1 had indicated a manageable safety and lasting response in advanced hepatocellular carcinoma.^7^ So far, there is no research which has constructed a prognosis signature by using immune‐related gene.

In this study, based on immune‐related genes from the ImmPort database, we used two RNA‐seq datasets from The Cancer Genome Atlas (TCGA) and the International Cancer Genome Consortium (ICGC) and one microarray dataset (http://www.ncbi.nlm.nih.gov/geo/query/acc.cgi?acc=GSE14520) to establish and validate a 33‐immune‐related gene pair signature for hepatocellular carcinoma patients. Then, we investigated the relationship between clinicalpathological factors and the prognostic signature. Finally, we compared this signature with other existing prognostic signatures to prove the predictive effectiveness and accuracy of this signature.

## 2 METHODS

### 2.1 Data source

The level‐three RNA‐seq expression data and clinical data of 377 HCC patient samples were downloaded from the TCGA data portal (https://portal.gdc.cancer.gov); patients with an overall survival time less than one month were excluded, and the dataset was randomly split into a training dataset (n = 206) and a testing dataset (n = 106). Another RNA‐seq dataset (n = 207) was downloaded from ICGC, and a microarray dataset (http://www.ncbi.nlm.nih.gov/geo/query/acc.cgi?acc=GSE14520) downloaded from the GEO database (http://www.ncbi.nlm.nih.gov/geo) was used as a dataset for validation of the signature. We downloaded 1534 immune‐related genes from the ImmPort database (https://immport.niaid.nih.gov). The immune‐related genes included cytokines, cytokine receptors, and genes correlated with the T‐cell receptor and B‐cell antigen receptor signaling pathways, natural killer cell cytotoxicity, and the antigen processing and presentation pathways.

### 2.2 Data preprocessing

When multiple probes matched the same target gene, the average expression value of the probes was used to represent the single gene expression level. When a patient had more than one sample, the average expression value of each gene was used to represent the level of gene expression in the patient.

### 2.3 Establishment of the prognostic signature based on immune‐related genes

A pairwise comparison was performed between the immune‐related gene expression value in each sample to obtain a score for each IRGP. If the expression level of the first IRG was higher than that of the second IRG in a specific IRGP, the score of this IRGP was 1; otherwise, the score was 0. If the score of an IRGP was 0 or 1 in more than 90% of the samples of the TCGA training dataset or the TCGA testing dataset, then we discarded the IRGP. The log‐rank test was applied to select the prognostic IRGPs (FDR < 0.01) in the training dataset, and then Lasso penalized Cox regression (iteration = 1000) was applied to generate a more stable prognostic gene model by using an R package (glmnet, version: 2.0‐16). The tuning parameter was estimated in the training dataset by performing 10‐fold cross‐validation. The most stable gene pair model was used to construct the prognostic signature, and then patients were assigned into high immune risk and low immune risk groups according to an immune risk cutoff score; the median value of the risk score was set as the cutoff value.

### 2.4 Validation and assessment of the IRGP signature

To validate the IRGP signature, the risk score was calculated according to the prognostic signature in every testing dataset; then, we assigned patients into low immune risk and high immune risk groups according to the median value of the risk score. The overall survival difference between the low immune risk and high immune risk groups was evaluated by the log‐rank test and Cox regression analysis. In addition, we compared the prognostic signature with three existing gene prognostic signatures by the receiver operating characteristic curve (ROC) curve and c‐index analyses in the full TCGA dataset.

### 2.5 Gene set enrichment analysis

To understand the underlying biological mechanisms of this immune‐related prognostic signature, we performed gene set enrichment analysis by using the MSigDB hallmark gene set (http://software.broadinstitute.org/gsea/downloads.jsp). An FDR value below 0.25 was considered statistically significant.

### 2.6 Statistical analysis

All statistical analyses were performed using GraphPad Prism 6 and R software (version 3.5.1, https://www.r-project.org/). The log‐rank test was used to evaluate the relationship between IRGPs and overall survival. The survival curves were generated by the R package “survminer”. The gene model was conducted with the “glmnet” package. The ROC curves were conducted by an R package called “survivalROC”. The c‐index was calculated by the R package “survcomp”.

## 3 RESULTS

### 3.1 Construction and definition of the IRGP signature

**Figure 1:**
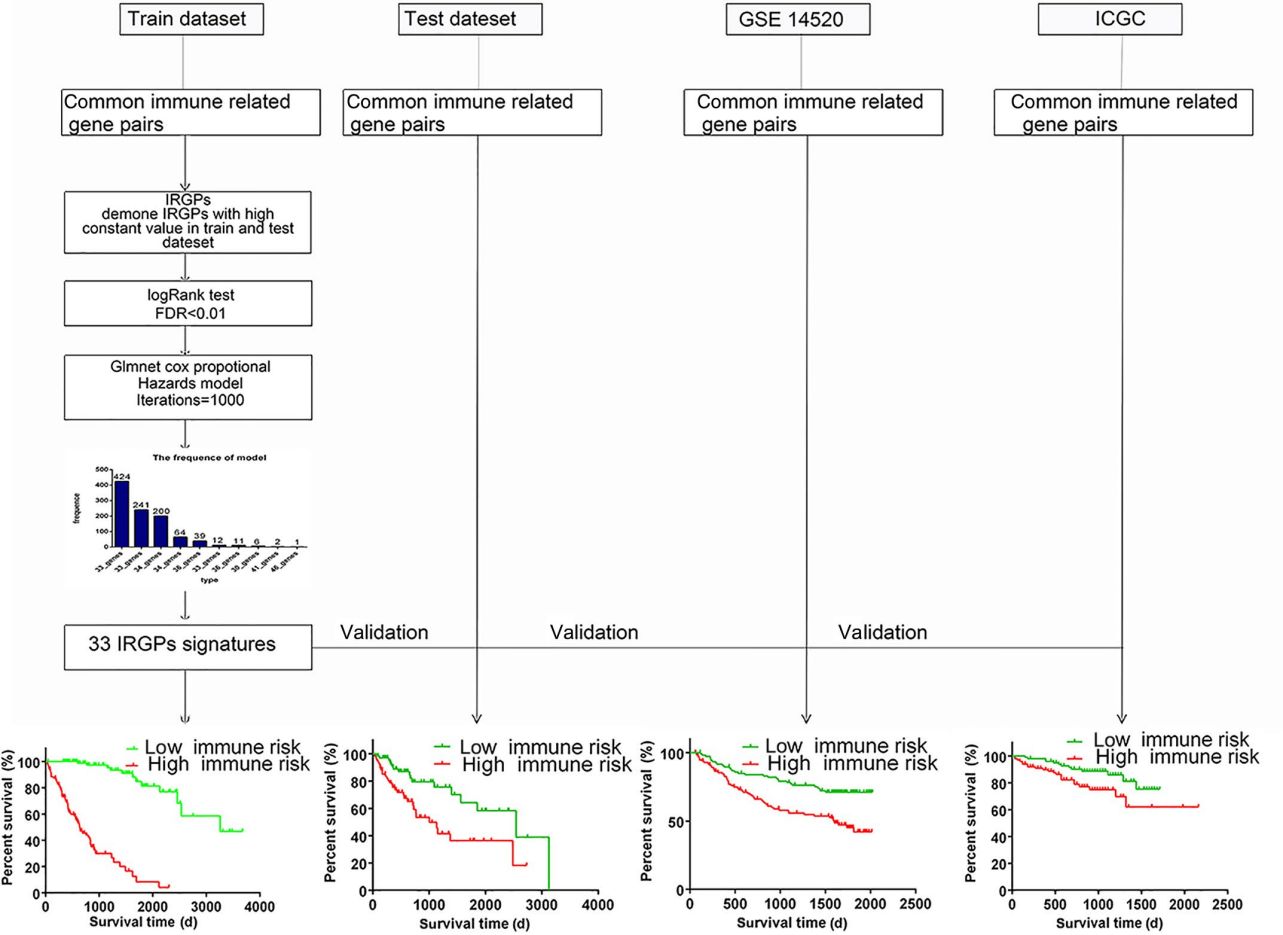
The workflow describes the construction and validation of our 33 IRGPs. The TCGA data were assigned into a training dataset (206) and a testing dataset (136), and the training dataset was used to construct immune‐related gene pair signatures. The testing, http://www.ncbi.nlm.nih.gov/geo/query/acc.cgi?acc=GSE14520 and ICGC datasets were used to validate the 33‐immune‐related gene pair signature

**Figure 2:**
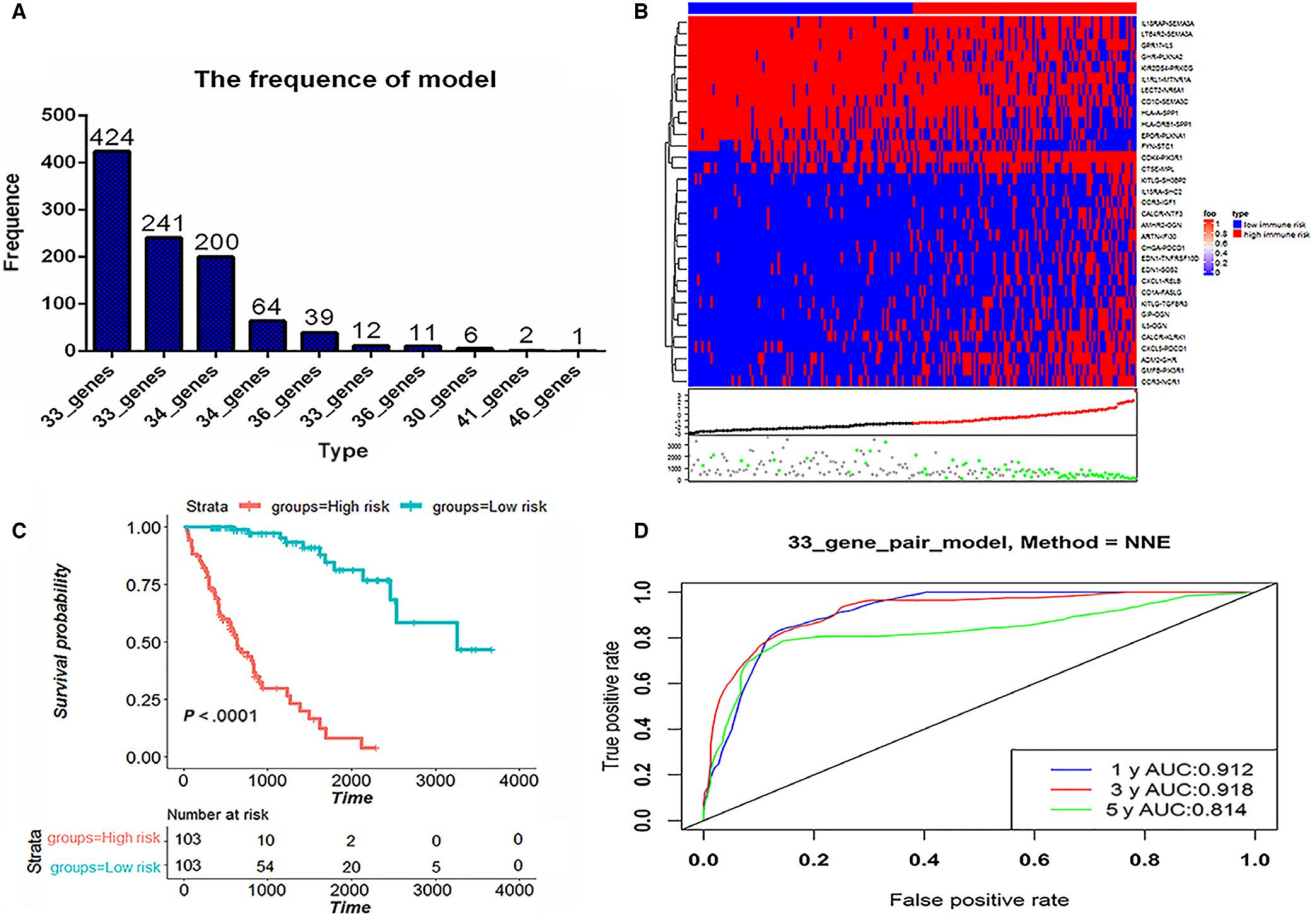
Construction and definition of IRGP signature. A, After 1000 iterations, the 33‐IRGP model achieved the highest frequency compared with the other nine IRGP models. The 33‐IRGP model was selected to construct the IRGP signature. B, The heatmap shows the score of the 33 IRGPs according to patient risk score. The patients were divided into high immune risk and low immune risk groups according to the median risk score. The red and black points represent the risk scores of high‐risk group patients and low‐risk group patients, respectively. The gray and green points represent patients who were alive or dead, respectively. C, The survival curve shows that high‐risk group patients had a poorer outcome than low‐risk group patients in the training dataset (*P* < .05). D, Generation of receiver operating characteristic (ROC) curves illustrated the predictive ability of the 33‐immune‐related gene pair model. The areas under the curves for 1‐, 3‐, and 5‐year survival were 0.912, 0.918, and 0.814, respectively, in the training dataset

To make our investigation procedure clearer, the entire workflow is illustrated in Figure 1. As shown in Table 1, a total of 765 HCC patients were included in our study. The TCGA dataset was randomly split into a training dataset (n = 206) and a testing dataset (n = 136). A total of 822 immune‐related genes were common among all datasets, and 337 431 IRGPs were constructed. Ultimately, we kept 99 615 IRGPs after removing IRGPs with a score of 0 or 1 in more than 90% of the samples in the TCGA training or testing datasets. Using the log‐rank test, we selected 188 prognostic IRGPs that were significantly associated with patient overall survival (FDR < 0.01). Next, the prognostic IRGPs were used to construct prognostic gene models by using Lasso penalized Cox regression on the TCGA training dataset. After 1000 iterations, the 33‐gene model, which had the highest frequency of (424) compared with the other nine gene models (Table S1), was used to construct the prognostic signature (Figure 2A). The 33‐IRGP prognostic signature information is shown in Table 2. The 33 IRGPs could accurately predict patient prognosis in the training dataset (Figure S1). The area under the receiver operating characteristic curve (AUC) values of the 1‐, 3‐, and 5‐year survival rates were 0.912, 0.918, and 0.816, respectively, in the training dataset (Figure 2D), which demonstrated that the predictive ability of our IRGP prognostic signature was promising. In the training dataset, the risk score of each patient was calculated with the immune prognostic signature, and then patients were assigned into low immune risk and high immune risk groups according to the median risk score. As shown in Figure 2C, the high immune risk group had a poorer prognosis than the low immune risk group (HR: 10.89, 95%CI: 8.09‐21.07, *P* < .0001) in the training dataset. We also found consistent results in the subgroup analysis (Table 3, Figure 2).

**Table 1:** Clinical and pathologic factors of the datasets used in this study

	TCGA (n, %)	ICGC (n, %)	http://www.ncbi.nlm.nih.gov/geo/query/acc.cgi?acc=GSE14520 (n, %)
Age			
<60	66 (19.3%)	39 (19.3%)	178 (80.5%)
≥60	276 (80.7%)	163 (80.7%)	43 (19.5%)
Gender			
Female	109 (31.9%)	50(24.8%)	30(13.6%)
Male	233 (68.1%)	152 (75.2%)	191 (86.4%)
Virus infection			
Yes	142(41.5%)	173 (85.7%)	212 (96%)
No	200(58.5%)	29 (14.3%)	6 (2.7%)
NA			3 (1.3%)
Cirrhosis			
Yes	127 (37%)	193(95.5%)	18(8.1%)
No	72 (21%)	9(4.5%)	203(91.9%)
NA	143 (42%)		
Recurrence			
Yes	173 (50.6%)		121 (54.8%)
No	125 (36.5%)		100 (45.2%)
NA	44 (12.9%)		
TNM stage			
Stage I	103		93 (42.1%)
Stage II	39		77 (34.8%)
Stage III	47		49 (22.2%)
Stage IV	2		2 (0.9%)
NA			0
Survival status			
Alive	219 (64.0%)	167 (82.7%)	136 (61.5%)
Dead	123 (36%)	35 (17.3%)	85 (38.5%)
Median follow‐up time(mo)	20.745 (1.02‐120.7)	27 (1‐72)	52.3 (2‐67.4)

Abbreviations: ICGC, ICGC LIHC dataset; NA represents information not available; TCGA, TCGA LIHC dataset.

**Table 2:** Information on the 33 IRGPs

Gene pair1	Full name		Gene pair2	Full name	Coefficient
ADM2		Adrenomedullin 2	GHR	Growth hormone receptor	0.094222
AMHR2		Anti‐Mullerian hormone receptor, type II	OGN	Osteoglycin	0.000652
ARTN		Artemin	IFI30	Interferon, gamma‐inducible protein 30	0.89792
CALCR		Calcitonin receptor	KLRK1	Killer cell lectin‐like receptor subfamily K, member 1	0.472908
CALCR		Calcitonin receptor	NTF3	Neurotrophin 3	0.110016
CCR3		Chemokine (C‐C motif) receptor 3	IGF1	Insulin‐like growth factor 1 (somatomedin C)	0.025693
CCR3		Chemokine (C‐C motif) receptor 3	NCR1	Natural cytotoxicity triggering receptor 1	0.070971
CD1A		CD1a molecule	FASLG	CD1a molecule	0.231429
CD1C		CD1c molecule	SEMA3C	CD1c molecule	−0.32233
CDK4		Cyclin‐dependent kinase 4	PIK3R1	Cyclin‐dependent kinase 4	0.489564
CHGA		Chromogranin A (parathyroid secretory protein 1)	PDCD1	Chromogranin A (parathyroid secretory protein 1)	0.483017
CTSE		Cathepsin E	MPL	Cathepsin E	0.152814
CXCL1		Chemokine (C‐X‐C motif) ligand 1 (melanoma growth stimulating activity, alpha)	RELB	Chemokine (C‐X‐C motif) ligand 1 (melanoma growth stimulating activity, alpha)	0.082072
CXCL5		Chemokine (C‐X‐C motif) ligand 5	PDCD1	Programmed cell death 1	0.031422
EDN1		Endothelin 1	SOS2	Son of sevenless homolog 2 (Drosophila)	0.164111
EDN1		Endothelin 1	TNFRSF10D	Tumor necrosis factor receptor superfamily, member 10d, decoy with truncated death domain	0.299299
EPOR		Erythropoietin receptor	PLXNA1	Plexin A1	−0.11661
FYN		FYN oncogene related to SRC, FGR, YES	STC1	Stanniocalcin 1	−0.37861
GHR		Growth hormone receptor	PLXNA2	Plexin A2	−0.03843
GIP		Gastric inhibitory polypeptide	OGN	Osteoglycin	0.227285
GMFB		Glia maturation factor, beta	PIK3R1	Phosphoinositide‐3‐kinase, regulatory subunit 1 (alpha)	0.003817
GPR17		G protein‐coupled receptor 17	IL5	Interleukin 5 (colony‐stimulating factor, eosinophil)	−0.14056
HLA‐A		Major histocompatibility complex, class I, A	SPP1	Secreted phosphoprotein 1	−0.04005
HLA‐DRB1		major histocompatibility complex, class II, DR beta 1	SPP1	Secreted phosphoprotein 1	−0.14776
IL15RA		Interleukin 15 receptor, alpha	SHC2	SHC (Src homology 2 domain containing) transforming protein 2	0.455341
IL18RAP		Interleukin 18 receptor accessory protein	SEMA3A	Sema domain, immunoglobulin domain (Ig), short basic domain, secreted, (semaphorin) 3A	−0.35545
IL1RL1		Interleukin 1 receptor‐like 1	MTNR1A	Melatonin receptor 1A	−0.56454
IL5		Interleukin 5 (colony‐stimulating factor, eosinophil)	OGN	Osteoglycin	0.361037
KIR2DS4‐			PRKCG	Protein kinase C, gamma	−0.49709
KITLG		KIT ligand	SH3BP2	SH3‐domain binding protein 2	0.035557
KITLG		KIT ligand	TGFBR3	Transforming growth factor, beta receptor III	0.107891
LECT2		Leukocyte cell‐derived chemotaxin 2	NR6A1	Leukocyte cell‐derived chemotaxin 2	−0.24611
LTB4R2		Leukotriene B4 receptor 2	SEMA3A	Leukotriene B4 receptor 2	−0.1266

**Table 3:** Clinical subgroup analysis of prognosis based on our IRGP signature

Variable	No. of patients		HR(95%CI)	Log‐rank *P*‐value
	Low risk	High risk		
All	103	104	10.89 (8.09‐21.07)	<.0001
Age				
Age < 60	25	13	29.17 (10.05 −186.3)	<.0001
Age ≥ 60	78	90	8.99 (8.090 −21.07)	<.0001
Gender				
Female	35	35	6.03 (4.243 −19.85)	<.0001
Male	68	68	19.95 (8.572 −28.92)	<.0001
TNM				
StageI/Ⅱ	87	63	13.88 (12.29 −48.39)	<.0001
StageⅢ/Ⅳ	15	40	5.32 (1.947 −7.748)	.0002
Grade				
G1/G2	73	59	9.05 (8.245 −28.29)	<.0001
G3/G4	29	43	10.35 (4.637 −23.11)	<.0001
Viral infection				
No	52	70	8.44 (5.296 ‐ 15.38)	<.0001
Yes	51	33	14.40 (7.895 −59.81)	<.0001
Recurrence	46	62	7.19 (4.375 −13.85)	<.0001

Abbreviations: All, TCGA LIHC dataset; CI, confidence interval; HR, hazard ratio.

### 3.2 Validation of the IRGP signature

**Figure 3:**
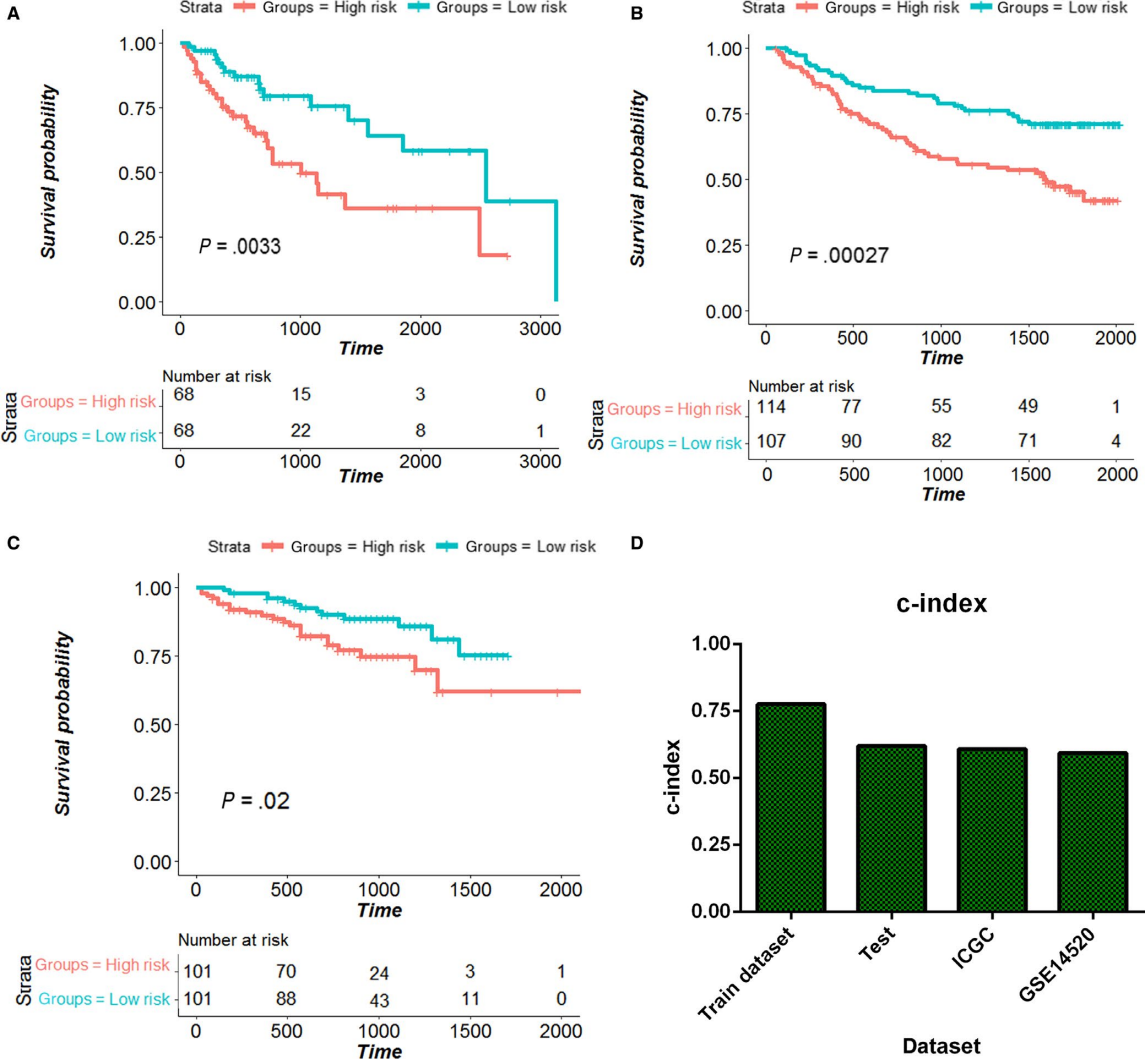
Validation of the IRGP signature. As shown, patients with a high risk score have a worse overall survival rate than those in the low risk score group according to Kaplan–Meier survival analysis in the TCGA test dataset (A), http://www.ncbi.nlm.nih.gov/geo/query/acc.cgi?acc=GSE14529 dataset (B), and ICGC dataset (C). These results show that the 33‐IRGP model has a robust predictive ability (*P* < .05). D: The c‐index values for the training dataset, testing dataset, http://www.ncbi.nlm.nih.gov/geo/query/acc.cgi?acc=GSE14520 dataset, and ICGC dataset were 0.78, 0.62, 0.59, and 0.61, respectively

In the TCGA, ICGC, and http://www.ncbi.nlm.nih.gov/geo/query/acc.cgi?acc=GSE14520 datasets, the risk score of each patient was calculated with the same 33‐IRGP prognostic signature, and patients were assigned into low immune risk and high immune risk groups according to the median risk score. The high immune risk group had poorer OS in all datasets than the low immune risk group (Figure 3A‐C). The c‐index values for the training, testing, ICGC and http://www.ncbi.nlm.nih.gov/geo/query/acc.cgi?acc=GSE14520 datasets were 0.78, 0.62, 0.61, and 0.59, respectively (Figure 3D). The multivariate Cox regression analysis showed that the IRGP risk score was an independent prognostic factor after adjustment for by age, sex, and TNM stage in the training (HR: 20.59, 95%CI: 8.73‐48.54, *P* = .000), testing (HR: 2.07, 95%CI: 1.07‐4.015, *P* = .031), http://www.ncbi.nlm.nih.gov/geo/query/acc.cgi?acc=GSE14520 (HR: 1.77, 95%CI: 1.09‐2.87) and ICGC datasets (HR: 2.40, 95%CI: 1.19‐4.82).

### 3.3 COMPARISON WITH OTHER PUBLISHED PROGNOSTIC SIGNATURES

**Figure 4:**
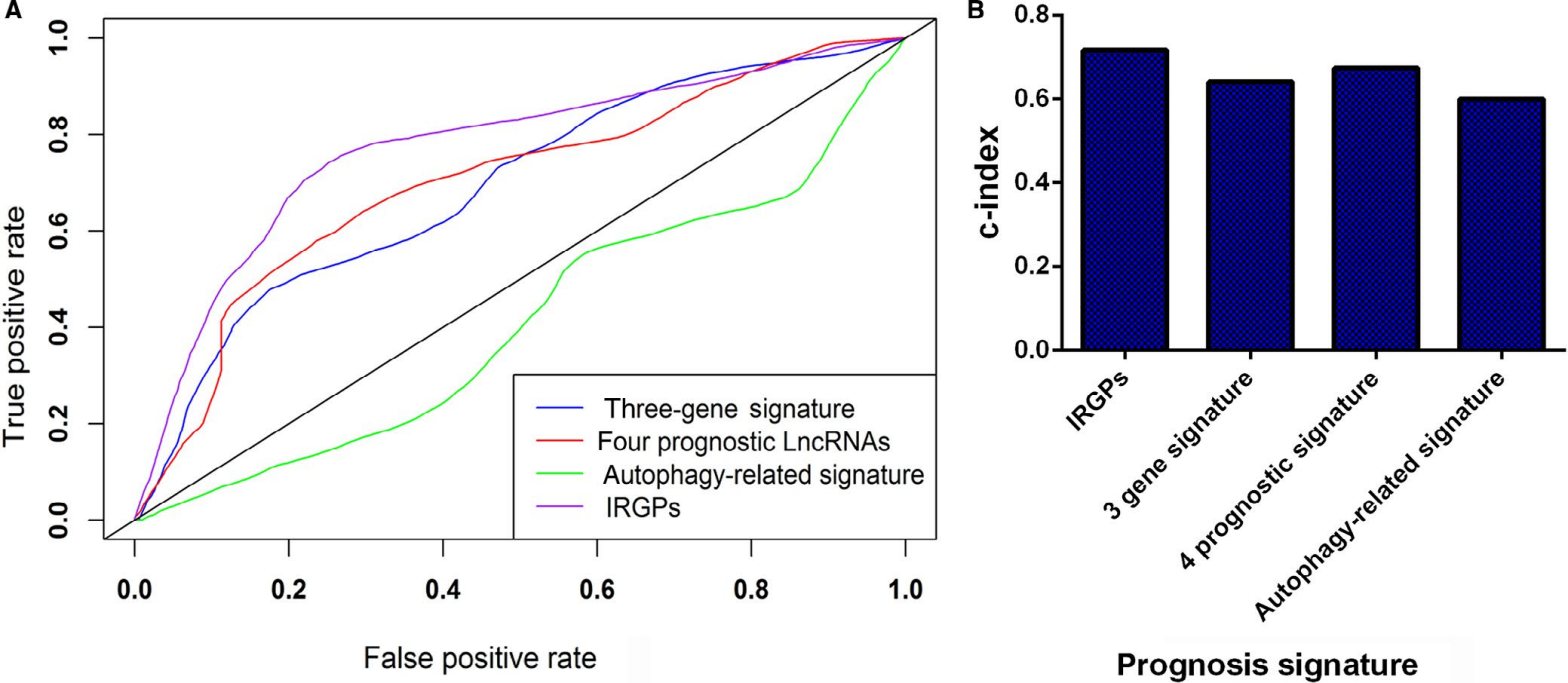
Determination of the receiver operating characteristic (ROC) curve (A) and c‐index (B) for different prognostic signatures. The AUC values for the IRGP model, three‐gene model, four prognostic lncRNA model, and autophagy‐related signature were 0.772, 0.691, 0.702, and 0.408, respectively. The c‐index values for the IRGP model, three‐gene model, four prognostic lncRNA model, and autophagy‐related signature were 0.772, 0.691, 0.702, and 0.408, respectively. These results indicate that our signature possesses a higher predictive efficacy and accuracy than the other models

We also compared our IRGP prognostic signature with three published gene prognostic signatures^8^, ^9^, ^10^ by constructing an ROC curve for 5‐year OS and determining the c‐index, and all data came from TCGA. As shown in Figure 4A‐B and Table 4, the AUC was 0.772 and the c‐index was 0.717 for our prognostic signature, and the IRGP prognostic signature possessed a higher predictive efficacy and accuracy than the existing three‐gene prognostic signature (AUC = 0.691, c‐index = 0.641), the 4‐gene prognostic signature (AUC = 0.702, c‐index = 0.674) and the autophagy‐related signature (AUC = 0.408, c‐index = 0.600) (Table 5).

**Table 4:** Multivariate Cox analysis of clinicopathological factors and risk signatures

Variable	HR	95%CI	*P*‐value
Training dataset			
Risk_score (low risk vs high risk)	20.59	8.73‐48.54	.000
Age (<60 vs ≥60)	1.17	0.58‐2.36	.662
Stage (I and II vs III and IV)	1.77	1.06‐2.97	.029
Gender (male vs female)	1.16	0.70‐1.91	.574
Testing dataset			
Risk_score	2.07	1.07‐4.015	.031
Age(<60 vs ≥60)	0.68	0.34‐1.38	.289
Stage (I and II vs III and IV)	2.27	1.21‐4.23	.010
Gender (male vs female)	1.61	0.859‐3.02	.138
ICGC dataset			
Risk_score	2.40	1.19‐4.82	.014
Age (<60 vs ≥60)	0.955	0.413‐2.21	.913
Gender (male vs female)	0.481	0.24‐0.98	.045
http://www.ncbi.nlm.nih.gov/geo/query/acc.cgi?acc=GSE14520 dataset			
Risk_score	1.77	1.09‐2.87	.022
Age (<60 vs ≥60)	1.05	0.58‐1.90	.868
Stage (I and II vs III and IV)	2.78	1.71‐4.50	.00
Gender (male vs female)	1.37	0.65‐2.87	.408

Abbreviations: CI, confidence interval; HR, hazard ratio.

**Table 5:** c‐index and AUC values between different signatures

Signature	AUC	c‐index
IRGPs	0.772	0.717
3 gene signature	0.691	0.641
4 prognostic signature	0.702	0.674
Autophagy‐related signature	0.408	0.600

Abbreviations: AUC, area under the receiver operating characteristic (ROC) curve; c‐index, concordance index.

### 3.4 Biological processes correlated with the IRGP signature

**Figure 5:**
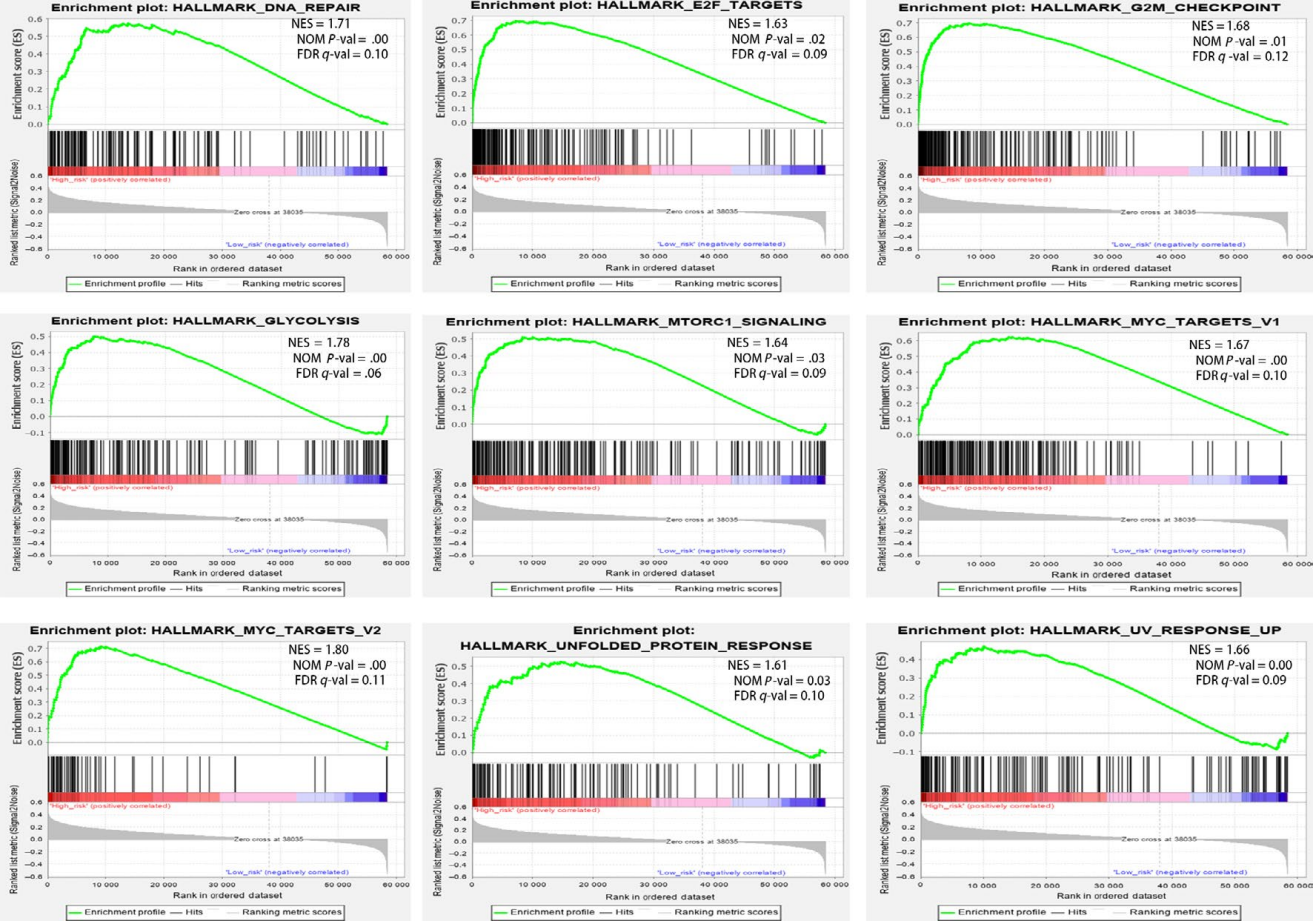
Gene set enrichment analysis (GSEA) between high and low immune risk groups. The results show that nine cancer hallmark gene sets are enriched in the high immune risk group in patients with HCC (*P* < .05, FDR < 0.25)

We assigned patients into low immune risk groups and high immune risk groups, and gene set enrichment analysis (GSEA) was performed on the training dataset. The result illustrated that a total of nine cancer hallmark gene sets were identified in the high‐risk group (Figure 5) including “MYC_TARGETS,” “GLYCOLYSIS,” and “DNA_REPAIR,” which indicated that these hallmark gene sets played a critical role in HCC progression.

## 4 DISCUSSION

It is well‐known that the liver participates in self‐tolerance and contains the richest immune effectors in the body.^11^ Several components of the immune system, including immune cells, chemokines, cytokines, and inhibitory receptors and ligands, have been shown to be key factors during tumor development and progression.^5^, ^6^ The complex immune environment of the liver makes immunotherapy a promising yet complicated strategy for treatment. There has also been a rapid rise in the amount of immunotherapy clinical trials in HCC in the past 15 years.^12^, ^13^, ^14^ Among these trials, immune checkpoint (PD1/PDL1 and CTLA‐4) blockade therapy has received great acclaim. Nivolumab (anti‐PD1) was the first FDA‐approved immune checkpoint inhibitor for HCC. In phase I and phase II clinical trials, 20% of HCC patients treated with nivolumab had a lasting response. In addition, several clinical trials of immune checkpoint inhibitors have also shown exciting results. HCC, like many other tumors, has an immunosuppressive microenvironment that can inhibit the activation of immune effectors, making adoptive immunotherapy a promising method. Recently, adoptive immunotherapies, including CIK cells, NK cells, NKT cells and CAR T cells, accounted for approximately half of the immunotherapy clinical trials in HCC (12 trials), and several studies have reported that adoptive immunotherapy can delay recurrence and prolong survival time.^15^, ^16^, ^17^ Cancer vaccines are another immunotherapy that can help the immune system recognize and attack cancer cells. Unfortunately, current vaccine monotherapies do not generate significant clinical outcomes in patients with HCC.^18^ In summary, immunotherapy is a promising treatment approach in HCC, and the immunology of hepatocellular carcinoma needs to be further explored. So, it is necessary to construct a prognostic signal using immune‐related genes.

Traditional prognostic signatures require the preprocessing of gene expression profiles, and this is a major factor that influences other widely used models. In this study, because our IRGPs were generated by pairwise comparison and the score was calculated entirely based on gene expression in the same patient, our prognostic signature can not only overcome the batch effects of the different platforms but also does not require the scaling and normalization of data. This approach has been reported to be robust in several studies, including cancer‐related studies, and it is a major advantage in our study.^19^, ^20^

In this study, by using Lasso penalized Cox regression, we constructed a 33‐IRGP prognostic signature and validated this signature in several different datasets. The results showed that our signature could stratify patients into high immune risk and low immune risk groups. Univariate and multivariate Cox proportional hazard regression analyses showed that the score was an independent prognostic factor. In our study, unlike in traditional studies, the signature was constructed by using Lasso penalized regression, which can identify the most suitable of many variables. Moreover, our signature was validated by several datasets, including RNA‐seq and microarray datasets. Finally, compared with the other three existing prognostic signatures, our signature possesses a higher predictive efficacy and accuracy.^8^, ^9^, ^10^

Our 33‐IRGP signature consists of 54 immune‐related genes, and these genes are mainly involved in the functions of immune cells and antigen identification and presentation and they play an important role in the composition of the immune microenvironment. CXCL5 and CXCL1 can promote intratumoral neutrophil infiltration, and their overexpression has been correlated with poor prognosis in HCC.^21^, ^22^, ^23^ CDK4 is a promising anticancer target in several cancers, including hepatocellular carcinoma. Shom Goel et al recently found that CDK4/6 inhibitors could promote tumor immunogenicity and may have synergistic effects with immunotherapy.^24^, ^25^ It was reported that the downregulation of LECT2 fostered the accumulation of inflammatory monocytes, which harbor immunosuppressive properties, and promoted the progression of hepatocellular carcinoma.^26^ PD1 is mainly expressed on effector T cells in tumor tissues in HCC. Compared with cirrhotic tissue, tumor tissue has a higher number of PD‐1+CD8+ T cells. Moreover, patients with higher levels of tumor‐infiltrating and circulating PD‐1+CD8+ T cells tend to progress early after posthepatic resection.^27^ It has been reported that macrophages can be recruited into HCC tissue by SEMA3A, and overexpression of SEMA3A indicates poor prognosis in hepatocellular carcinoma.^28^ Artemin was shown to be related to early relapse, shortened overall survival and large tumor size.^29^ The involvement of all the above mentioned genes indicates that immune processes contribute to tumor development and prognosis. Other immune‐related genes in our signature can also predict the prognosis of HCC patients. In addition, expression imbalances in certain gene pairs may play a more important role than individual differentially expressed genes. GSEA indicated that “MYC_TARGETS,” “DNA_REPAIR,” and “GLYCOLYSIS” were enriched in the high‐risk group, and these results were consistent with previous reports.^30^, ^31^, ^32^

Nevertheless, we should acknowledge the limitations of this study. First, our research was a retrospective analysis, and a prospective cohort is needed to validate the results. Second, because the signature was constructed by using immune‐related genes, our signature does not represent diverse biological processes. Finally, the signature was constructed by using RNA‐seq and microarray expression data. Further clinical applications should be evaluated by using RT‐PCR or IHC.

In conclusion, we developed a new IRGP prognostic model in HCC.

## CONFLICT OF INTEREST

The authors declare that no competing interest exists.

## Data Availability

TCGA dataset that supports the findings of this study is openly available in [University of California Santa Cruz (UCSC) Genome Browser], reference number [LIHC].
