# Supplementary figures and images for: A signature of 33 immune‐related gene pairs predicts clinical outcome in hepatocellular carcinoma
Source: Cancer Med. 2020 Feb 18;9(8):2868–78. doi: 10.1002/cam4.2921 (PMC7163092; doi:10.1002/cam4.2921)

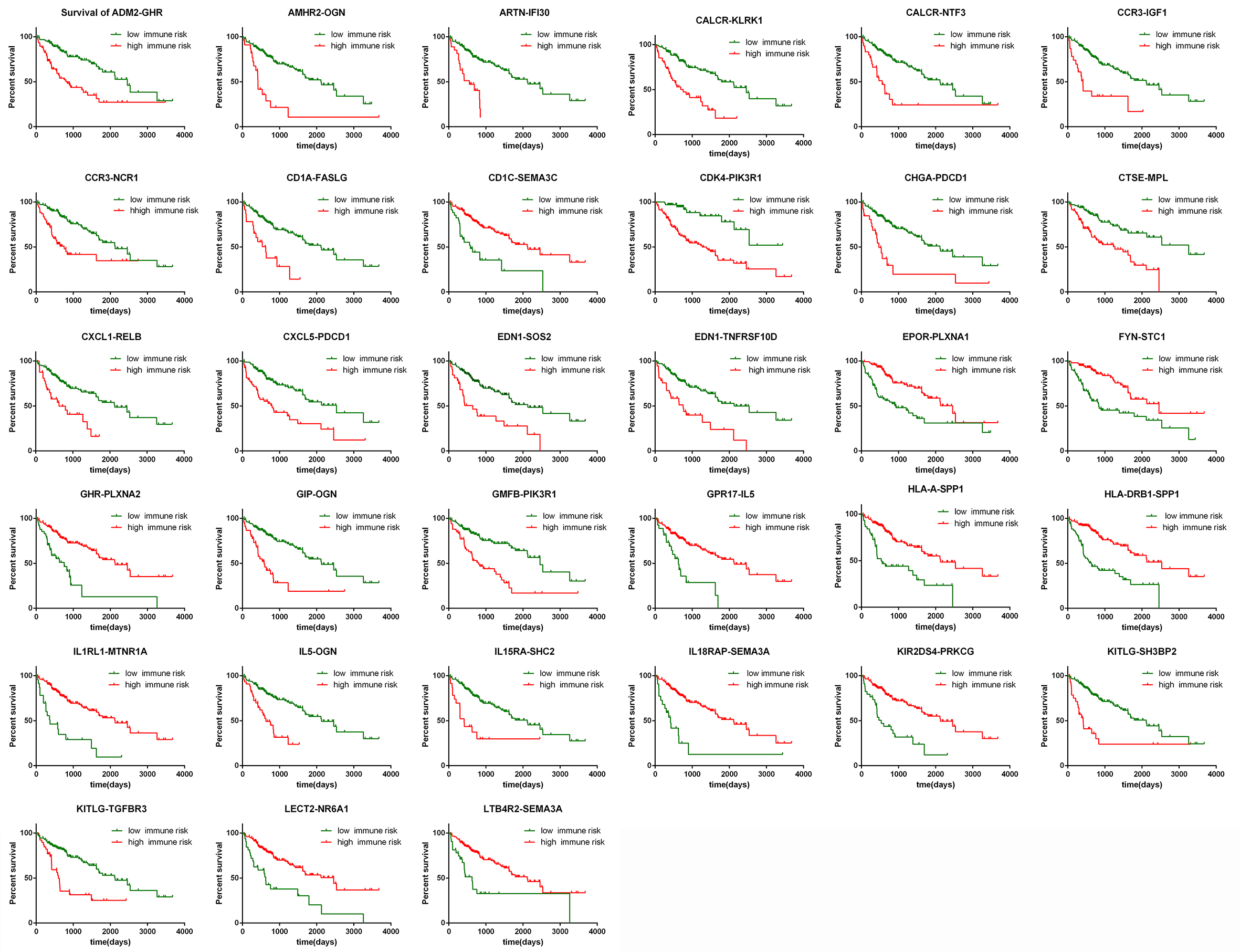

Supplement: Supplementary file 1 [file CAM4-9-2868-s001.tif]
