# Supplementary material for: A signature of 33 immune‐related gene pairs predicts clinical outcome in hepatocellular carcinoma
Source: Cancer Med. 2020 Feb 18;9(8):2868–78. doi: 10.1002/cam4.2921 (PMC7163092; doi:10.1002/cam4.2921)
Supplement: Supplementary file 2 [file CAM4-9-2868-s002.docx]

| Type  **TableS1**  **The information of all gene pairs model is shown on the table** | Gene pairs | frequency |
| --- | --- | --- |
| 1_33_gene pairs | ADM2-GHR;AMHR2-OGN;ARTN-IFI30;CALCR-KLRK1;CALCR-NTF3;CCR3-IGF1;CCR3-NCR1;CD1A-FASLG;CD1C-SEMA3C;CDK4-PIK3R1;CHGA-PDCD1;CTSE-MPL;CXCL1-RELB;CXCL5-PDCD1;EDN1-SOS2;EDN1-TNFRSF10D;EPOR-PLXNA1;FYN-STC1;GHR-PLXNA2;GIP-OGN;GMFB-PIK3R1;GPR17-IL5;HLA-A-SPP1;HLA-DRB1-SPP1;IL15RA-SHC2;IL18RAP-SEMA3A;IL1RL1-MTNR1A;IL5-OGN;KIR2DS4-PRKCG;KITLG-SH3BP2;KITLG-TGFBR3;LECT2-NR6A1;LTB4R2-SEMA3A | 424 |
| 2_34_gene pairs | ADM2-GHR;ARTN-IFI30;CALCR-KLRK1;CALCR-NTF3;CCR3-IGF1;CCR3-NCR1;CD1A-FASLG;CD1C-SEMA3C;CDK4-PIK3R1;CHGA-PDCD1;CTSE-MPL;CXCL1-RELB;CXCL5-PDCD1;EDN1-SOS2;EDN1-TNFRSF10D;EPOR-PLXNA1;FYN-STC1;GHR-KITLG;GHR-PLXNA2;GIP-OGN;GMFB-PIK3R1;GPR17-IL5;HLA-A-SPP1;HLA-DRB1-SPP1;IL15RA-SHC2;IL18RAP-SEMA3A;IL1RL1-MTNR1A;IL5-OGN;KIR2DS4-PRKCG;KITLG-SH3BP2;KITLG-TGFBR3;LECT2-NR6A1;LHB-MC1R;LTB4R2-SEMA3A | 64 |
| 3_36_gene pairs | ADM2-GHR;AMHR2-OGN;ARTN-IFI30;BCL10-HLA-DQB1;CALCR-KLRK1;CALCR-NTF3;CCR3-IGF1;CCR3-NCR1;CD1A-FASLG;CD1C-SEMA3C;CDK4-PIK3R1;CHGA-PDCD1;CTSE-MPL;CXCL1-RELB;CXCL5-PDCD1;EDN1-SOS2;EDN1-TNFRSF10D;EPOR-PLXNA1;FYN-STC1;GHR-PLXNA2;GIP-OGN;GPR17-IL5;HLA-A-SPP1;HLA-DRB1-SPP1;IL12A-PTGER2;IL12B-OGN;IL15RA-SHC2;IL18RAP-SEMA3A;IL1RL1-MTNR1A;IL5-OGN;KIR2DS4-PRKCG;KITLG-SH3BP2;KITLG-TGFBR3;LECT2-NR6A1;LTB4R2-SEMA3A;NCR3-NR0B1 | 11 |
| 4_33_gene pairs | ADM2-GHR;ARTN-IFI30;CALCR-KLRK1;CALCR-NTF3;CCR3-IGF1;CCR3-NCR1;CD1A-FASLG;CD1C-SEMA3C;CDK4-PIK3R1;CHGA-PDCD1;CTSE-MPL;CXCL1-RELB;CXCL5-PDCD1;EDN1-SOS2;EDN1-TNFRSF10D;EPOR-PLXNA1;FYN-STC1;GHR-KITLG;GHR-PLXNA2;GIP-OGN;GMFB-PIK3R1;GPR17-IL5;HLA-A-SPP1;HLA-DRB1-SPP1;IL15RA-SHC2;IL18RAP-SEMA3A;IL1RL1-MTNR1A;IL5-OGN;KIR2DS4-PRKCG;KITLG-SH3BP2;KITLG-TGFBR3;LECT2-NR6A1;LTB4R2-SEMA3A | 241 |
| 6_34_gene pairs | ADM2-GHR;ARTN-IFI30;BCL10-HLA-DQB1;CALCR-KLRK1;CALCR-NTF3;CCR3-IGF1;CCR3-NCR1;CD1A-FASLG;CD1C-SEMA3C;CDK4-PIK3R1;CHGA-PDCD1;CTSE-MPL;CXCL1-RELB;CXCL5-PDCD1;EDN1-SOS2;EDN1-TNFRSF10D;EPOR-PLXNA1;FYN-STC1;GDF3-OGN;GHR-PLXNA2;GIP-OGN;GPR17-IL5;HLA-A-SPP1;HLA-DRB1-SPP1;IL12A-PTGER2;IL15RA-SHC2;IL18RAP-SEMA3A;IL1RL1-MTNR1A;IL5-OGN;KIR2DS4-PRKCG;KITLG-SH3BP2;KITLG-TGFBR3;LECT2-NR6A1;LTB4R2-SEMA3A | 200 |
| 10_36_gene pairs | ADM2-GHR;ARTN-IFI30;BCL10-HLA-DQB1;CALCR-KLRK1;CALCR-NTF3;CCR3-IGF1;CCR3-NCR1;CD1A-FASLG;CD1C-SEMA3C;CDK4-PIK3R1;CHGA-PDCD1;CTSE-MPL;CXCL1-RELB;CXCL5-PDCD1;EDN1-SOS2;EDN1-TNFRSF10D;EPOR-PLXNA1;FYN-STC1;GDF3-OGN;GHR-PLXNA2;GIP-OGN;GPR17-IL5;HLA-A-SPP1;HLA-DRB1-SPP1;IL12A-PTGER2;IL12B-OGN;IL15RA-SHC2;IL18RAP-SEMA3A;IL1RL1-MTNR1A;IL5-OGN;KIR2DS4-PRKCG;KITLG-SH3BP2;KITLG-TGFBR3;LECT2-NR6A1;LTB4R2-SEMA3A;NCR3-NR0B1 | 39 |
| 35_33_gene pairs | ADM2-GHR;ARTN-IFI30;CALCR-KLRK1;CALCR-NTF3;CCR3-KLRD1;CCR3-NCR1;CD1A-FASLG;CD1C-SEMA3C;CDK4-PIK3R1;CHGA-PDCD1;CTSE-MPL;CXCL1-RELB;CXCL5-PDCD1;EDN1-SOS2;EDN1-TNFRSF10D;EPOR-PLXNA1;FYN-STC1;GHR-KITLG;GIP-OGN;GMFB-PIK3R1;GPR17-IL5;HLA-A-SPP1;HLA-DRB1-SPP1;IL15RA-SHC2;IL18RAP-SEMA3A;IL1RL1-MTNR1A;IL5-OGN;KIR2DS4-PRKCG;KITLG-SH3BP2;KITLG-TGFBR3;LECT2-NR6A1;LHB-MC1R;LTB4R2-SEMA3A | 12 |
| 77_30_gene pairs | ADM2-GHR;ARTN-IFI30;CALCR-KLRK1;CALCR-NTF3;CCR3-KLRD1;CD1A-FASLG;CD1C-SEMA3C;CDK4-PIK3R1;CHGA-PDCD1;CTSE-MPL;CXCL5-PDCD1;EDN1-SOS2;EDN1-TNFRSF10D;EPOR-PLXNA1;FYN-STC1;GHR-KITLG;GIP-OGN;GMFB-PIK3R1;GPR17-IL5;HLA-DRB1-SPP1;IL15RA-SHC2;IL18RAP-SEMA3A;IL1RL1-MTNR1A;IL5-OGN;KIR2DS4-PRKCG;KITLG-TGFBR3;LECT2-LTBP2;LECT2-NR6A1;LHB-MC1R;LTB4R2-SEMA3A | 6 |
| 94_41_genes | ADM2-GHR;AMHR2-OGN;ARTN-IFI30;BCL10-HLA-DQB1;CALCR-KLRK1;CALCR-NTF3;CCR3-IGF1;CCR3-ITK;CCR3-NCR1;CD1A-FASLG;CD1C-SEMA3C;CDK4-PIK3R1;CHGA-PDCD1;CMA1-GIP;CTSE-MPL;CXCL1-RELB;CXCL5-PDCD1;CXCL6-LECT2;EDN1-SOS2;EDN1-TNFRSF10D;EPOR-PLXNA1;FYN-STC1;GHR-PLXNA2;GIP-OGN;GNRH1-NR0B1;GPR17-IL5;HLA-A-SPP1;HLA-DRB1-SPP1;IL12A-PTGER2;IL12B-OGN;IL15RA-SHC2;IL18RAP-SEMA3A;IL1RL1-MTNR1A;IL5-OGN;KIR2DS4-PRKCG;KITLG-SH3BP2;KITLG-TGFBR3;LECT2-NR6A1;LTB4R2-SEMA3A;NCR3-NR0B1;SDC3-TNFRSF1B | 2 |
| 950_46_gene pairs | ADM2-GHR;AMHR2-OGN;ARTN-IFI30;BCL10-HLA-DQB1;CALCR-KLRK1;CALCR-NTF3;CCR3-IGF1;CCR3-ITK;CCR3-NCR1;CD1A-FASLG;CD1C-SEMA3C;CD79A-LHB;CDK4-PIK3R1;CHGA-PDCD1;CTSE-MPL;CXCL1-NR1H4;CXCL1-RELB;CXCL5-PDCD1;CXCL6-LECT2;EDN1-LECT2;EDN1-SOS2;EDN1-TNFRSF10D;EPOR-PLXNA1;FYN-STC1;GHR-PLXNA2;GIP-OGN;GNRH1-NR0B1;GPR17-IL5;HLA-A-SPP1;IL12A-PTGER2;IL12B-OGN;IL15RA-SHC2;IL18RAP-SEMA3A;IL1RL1-MTNR1A;IL1RN-OSMR;IL5-OGN;ITK-NR0B1;KIR2DS4-PRKCG;KITLG-SH3BP2;KITLG-TGFBR3;LECT2-LTBP2;LECT2-NR6A1;LTB4R2-SEMA3A;NCR3-NR0B1;NR0B1-OGN;SDC3-TNFRSF1B | 1 |
